# Targeting *Mycobacterium tuberculosis* response to environmental cues for the development of effective antitubercular drugs

**DOI:** 10.1371/journal.pbio.3001355

**Published:** 2021-07-28

**Authors:** Richard C. Lavin, Calvin Johnson, Yong-Mo Ahn, Kyle M. Kremiller, Matthew Sherwood, Jimmy S. Patel, Yan Pan, Riccardo Russo, Nathan J. MacGilvary, David Giacalone, Yuzo L. Kevorkian, Matthew D. Zimmerman, J. Fraser Glickman, Joel S. Freundlich, Shumin Tan

**Affiliations:** 1 Department of Molecular Biology and Microbiology, Tufts University School of Medicine, Boston, Massachusetts, United States of America; 2 Graduate Program in Molecular Microbiology, Graduate School of Biomedical Sciences, Tufts University, Boston, Massachusetts, United States of America; 3 Department of Pharmacology, Physiology, and Neuroscience, Rutgers University–New Jersey Medical School, Newark, New Jersey, United States of America; 4 Center for Discovery and Innovation, Hackensack Meridian Health, Nutley, New Jersey, United States of America; 5 Division of Infectious Disease, Department of Medicine and the Ruy V. Lourenco Center for the Study of Emerging and Re-emerging Pathogens, Rutgers University–New Jersey Medical School, Newark, New Jersey, United States of America; 6 High-Throughput and Spectroscopy Resource Center, The Rockefeller University, New York, New York, United States of America; Stanford University, UNITED STATES

## Abstract

Sensing and response to environmental cues, such as pH and chloride (Cl^−^), is critical in enabling *Mycobacterium tuberculosis* (Mtb) colonization of its host. Utilizing a fluorescent reporter Mtb strain in a chemical screen, we have identified compounds that dysregulate Mtb response to high Cl^−^ levels, with a subset of the hits also inhibiting Mtb growth in host macrophages. Structure–activity relationship studies on the hit compound “C6,” or 2-(4-((2-(ethylthio)pyrimidin-5-yl)methyl)piperazin-1-yl)benzo[d]oxazole, demonstrated a correlation between compound perturbation of Mtb Cl^−^ response and inhibition of bacterial growth in macrophages. C6 accumulated in both bacterial and host cells, and inhibited Mtb growth in cholesterol media, but not in rich media. Subsequent examination of the Cl^−^ response of Mtb revealed an intriguing link with bacterial growth in cholesterol, with increased transcription of several Cl^−^-responsive genes in the simultaneous presence of cholesterol and high external Cl^−^ concentration, versus transcript levels observed during exposure to high external Cl^−^ concentration alone. Strikingly, oral administration of C6 was able to inhibit Mtb growth in vivo in a C3HeB/FeJ murine infection model. Our work illustrates how Mtb response to environmental cues can intersect with its metabolism and be exploited in antitubercular drug discovery.

## Abstract 2

Responding to environmental cues such as pH and chloride is critical in enabling Mycobacterium tuberculosis to colonize its host. A chemical screen using an M. tuberculosis strain bearing a fluorescent reporter identifies a compound that perturbs the bacterial response to chloride and inhibits its growth in a murine infection model.

## Introduction

Tuberculosis, caused by the bacterium *Mycobacterium tuberculosis* (Mtb), is a major global health concern that results in 1.4 million deaths annually [1]. The local environments that Mtb encounters during establishment and continued chronic colonization of its host are highly varied, with differences arising in contexts ranging from lesion sublocations to immune-driven changes within a single host cell [2–10]. The ability of Mtb to recognize and adapt to local microenvironments is consequently critical for host colonization [11–16], and a key microenvironment that Mtb must sense and respond to is that of the macrophage phagosome, which serves as a major replicative niche for Mtb [7,9,17–19]. Normal phagosome maturation entails acidification of the compartment, and while Mtb prevents complete maturation of the macrophage phagosome [20,21], the slight acidification that does occur continues to function as a major environmental cue for Mtb; almost half of the bacterial gene expression changes normally observed upon macrophage infection is lost if phagosome acidification is inhibited by concanamycin A treatment [18]. We have since reported that chloride concentration ([Cl^−^]) within the phagosome is inversely related to pH, with [Cl^−^] increasing as pH decreases [9]. Further, Mtb is able to respond to changes in environmental [Cl^−^], with the gene transcription changes observed in the presence of high external [Cl^−^] overlapping significantly with that seen upon bacterial exposure to acidic pH [9]. Strikingly, the transcriptional response of Mtb to acidic pH and high [Cl^−^] is highly synergistic, reinforcing the role that Cl^−^ can play as an environmental cue for Mtb [9].

Critically, the ability of Mtb to respond to the ionic signals of pH and Cl^−^ is crucial for proper colonization of the host, with disruption of the two-component regulatory system PhoPR, which is essential for pH response and important for Cl^−^ response [9,19], leading to significant attenuation of Mtb growth in vivo [11,12]. Other abundant ions, such as potassium (K^+^), also play an important role in Mtb host adaptation, as disruption of the Mtb Trk K^+^ uptake system results in a dampening of Mtb response to acidic pH and high [Cl^−^], and attenuation of Mtb growth in vivo [16]. The vital role of Mtb sensing and response to ionic cues for successful host colonization thus raises the possibility of exploiting this facet of Mtb–host interactions in therapeutic approaches.

In our previous study identifying Cl^−^ as a novel environmental signal that Mtb responds to in synergy with pH, we had generated and characterized an *rv2390c′*::GFP reporter Mtb strain that fluoresces specifically in response to high [Cl^−^] and acidic pH [9]. Fluorescent reporter strains provide a unique tool for analysis of Mtb–host interactions in vivo and for discovery of varied aspects of Mtb biology in vitro [7,9,22–24]. One facet in which fluorescent reporter Mtb strains can be exploited in vitro is in the uncovering of small molecule compounds that perturb a particular Mtb transcriptional pathway or Mtb response to a given host signal. For example, screening of small molecule compound libraries with an *aprA′*::GFP and *hspX′*::GFP reporter, whose induction in response to acidic pH and hypoxia/nitric oxide are controlled by the Mtb two-component systems PhoPR and DosRST, respectively, has led to the identification of inhibitors of these transcriptional regulators [25,26]. In the context of Cl^−^ response, while the bacterial regulatory network underlying its control remains to be fully elucidated, the synergistic response of Mtb to Cl^−^ and pH and the heterogeneity of these environmental signals during in vivo infection underscore the importance that Cl^−^ sensing and response can have for Mtb in the process of host colonization. The *rv2390c′*::GFP reporter thus presents an opportunity to identify and develop new tools directed at dysregulating Mtb response to Cl^−^, to better understand this important yet understudied aspect of Mtb–host interactions.

Here, we use our *rv2390c′*::GFP reporter Mtb strain in a chemical screen of 50,816 compounds to identify small molecule compounds that dysregulate Mtb response to Cl^−^. A tertiary screen examining the effect of hit compounds on Mtb growth in host macrophages, coupled with pharmacokinetic (PK) characterization, led to focusing of follow-up studies on the hit compound termed “C6.” Structure–activity relationship (SAR) analysis identified the importance of the 2-ethylthio group in the ability of C6 to dysregulate Mtb Cl^−^ response and impair bacterial growth in host cells. C6 accumulates in both Mtb and host macrophages but does not affect bacterial growth in standard rich broth or affect macrophage phagosome maturation characteristics. Mtb growth in cholesterol media was inhibited by treatment with C6, and induction of the *rv2390c′*::GFP reporter in high [Cl^−^] conditions was intriguingly found to be further increased in the context of cholesterol media, suggesting an intersection between Mtb metabolism and its response to Cl^−^. Most strikingly, oral administration of C6 in a C3HeB/FeJ murine infection model resulted in significant reduction in bacterial load, indicating its utility both as a chemical probe and a starting point for the development of novel antitubercular drugs.

## Results

### Reporter-based chemical screen identifies compounds that modulate Mtb response to environmental chloride levels

**Fig 1:**
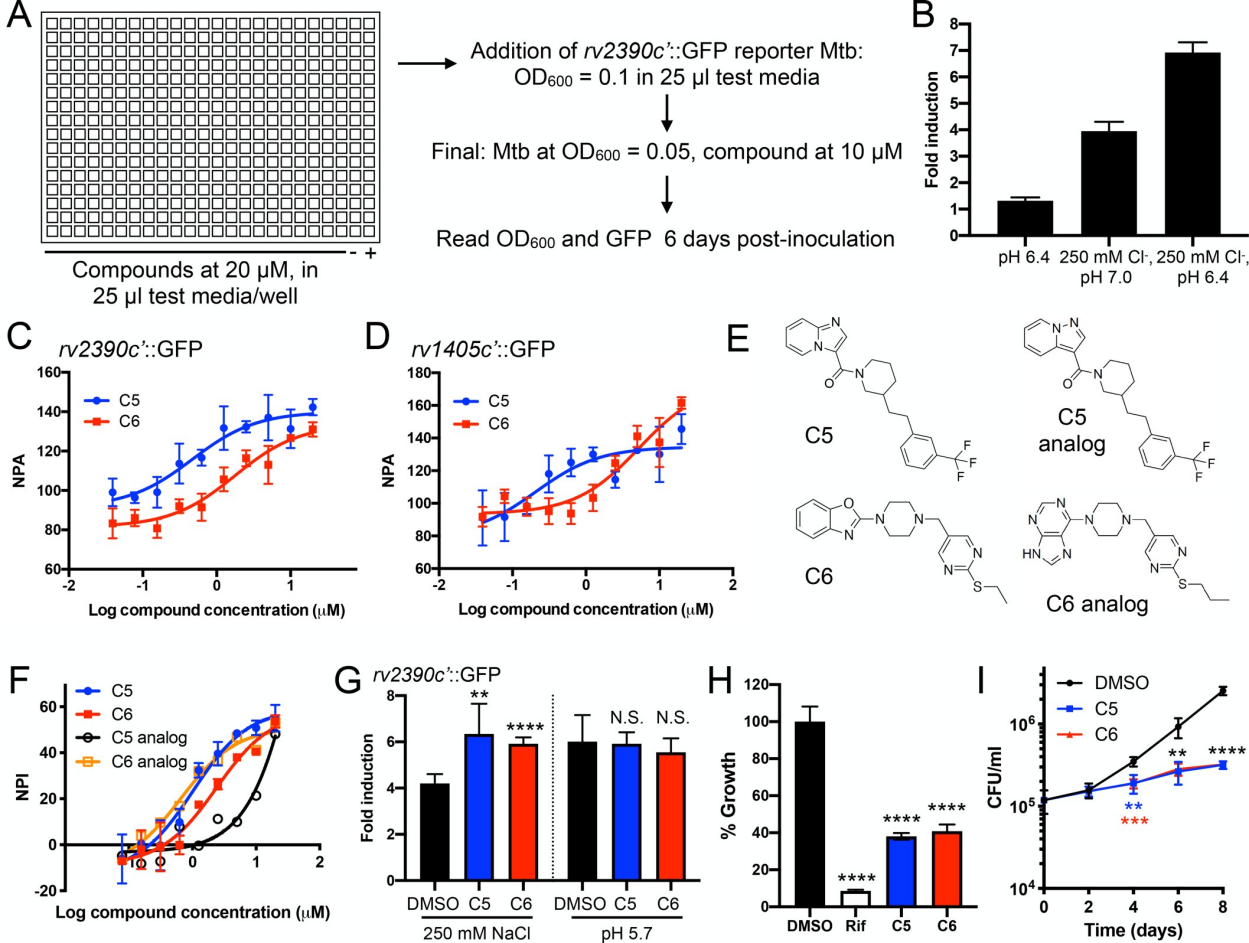
Reporter-based chemical screen identifies compounds that modulate Mtb response to environmental chloride levels and inhibit Mtb colonization of host macrophages. (A) *rv2390c′*::GFP screen set-up. Schematic of the compound screen conducted in 384-well plates. DMSO-treated control wells with Mtb in 7H9 (pH 6.4) media (negative, −) or 7H9 (pH 6.4), 250 mM NaCl media (positive, +) were included in each plate. (B) Dual high [Cl^−^], slightly acidic pH conditions provide increased reporter dynamic range. Mtb carrying the *rv2390c′*::GFP reporter was grown for 6 days in pH 7 control media or in the indicated conditions in a 384-well plate format. Fold induction represents *rv2390c*′::GFP signal/OD_600_ in each test condition as compared to the pH 7 control, measured by a microplate reader. Data are shown as means ± SD from 16 wells. (C) Dose response curve validation of 2 hit compounds. Mtb carrying the *rv2390c′*::GFP reporter was grown for 6 days in pH 6.4, 250 mM NaCl media treated with the indicated compound, in a 384-well plate format with controls as shown in the schematic in (A). NPA was calculated by setting GFP signal/OD_600_ of the reporter observed in the DMSO-treated positive control condition at 100% and comparing the compound-treated GFP signal/OD_600_ values to that baseline. Data are shown as means ± SD from 3 wells. The AC_50_ of compounds C5 and C6 was 0.5 μM and 1.8 μM, respectively. (D) Secondary screen results of 2 hit compounds. Mtb carrying the *rv1405c′*::GFP reporter was grown and assayed as in (C). Data are shown as means ± SD from 2–3 wells. (E) Structures of compounds C5 and C6 and their respective analogs in the screen. (F) Dose response curve testing of effect of hit compounds on Mtb growth in J774 macrophage-like cells. Mtb constitutively expressing mKO was used to infect J774 cells in a 384-well format, and cells treated with compounds, 5 μM rifampicin, or DMSO as a carrier control. mKO fluorescence was measured 6 days post-infection with a microplate reader. NPI was calculated by setting mKO signal observed in the rifampicin-treated condition as 100% inhibition (versus signal observed in the DMSO-treated controls) and comparing the mKO values in the compound-treated wells to that baseline. Data are shown as means ± SD from 2 wells. (G) Compounds C5 and C6 increase *rv2390c′*::GFP reporter response upon bacterial exposure to high [Cl^−^]. Mtb carrying the *rv2390c′*::GFP reporter was grown for 9 days in 7H9 (pH 7.0) ± 250 mM NaCl or 7H9 (pH 5.7), treated with DMSO as a carrier control, 10 μM C5 or 10 μM C6. Reporter signal in fixed samples was measure by flow cytometry, with fold signal induction compared to the corresponding treatment in the 7H9 (pH 7) control condition. Data are shown as means ± SD from 5 experiments. *p*-values were obtained with an unpaired *t* test, comparing each compound treatment to DMSO treatment for each condition. N.S., not significant, **p < 0.01, ****p < 0.0001. (H) C5 and C6 inhibit growth in J774 cells. J774 macrophage-like cells were infected with Mtb constitutively expressing mKO and treated with DMSO, 5 μM rifampicin, 10 μM C5, or 10 μM C6. Bacterial growth was tracked by fluorescence, with readings taken 6 days post-infection. DMSO is the carrier control, and growth in that condition is set at 100%. Data are shown as means ± SD from 3–4 wells. *p*-values were obtained with a one-way ANOVA with a Dunnett multiple corrections test, and treatment sets compared to the DMSO control. ****p < 0.0001. (I) C5 and C6 inhibit growth in primary BMDMs. BMDMs were infected with WT Mtb and bacterial load determined at indicated times. DMSO as a carrier control, 10 μM C5 or 10 μM C6 was added 2 hours post-infection. Data are shown as means ± SD from 4 wells, pooled from 2 independent experiments. *p*-values were obtained with an unpaired *t* test, comparing each treatment to the DMSO control for a given time point. *p*-value in blue and red correspond to those for C5 and C6, respectively, while those indicated in black apply to both C5 and C6. **p < 0.01, ***p < 0.001, ****p < 0.0001. The numerical data underlying the graphs shown in this figure are provided in S1 Data. AC_50_, activatory concentration, 50%; BMDM, bone marrow–derived macrophage; mKO, monomeric Kusabira Orange; Mtb, *Mycobacterium tuberculosis*; NPA, normalized percent activation; NPI, normalized percent inhibition; WT, wild-type.

To identify compounds that modulate Mtb response to environmental chloride (Cl^−^) levels, we exploited our previously described Cl^−^ and pH-responsive *rv2390c′*::GFP reporter Mtb strain in a 384-well format fluorescence-based screen (Fig 1A). GFP signal in this reporter strain is induced upon bacterial exposure to high Cl^−^ concentrations ([Cl^−^]) or acidic pH, with a synergistic response observed in the presence of both signals [9]. The test condition of 250 mM NaCl (pH 6.4) was chosen to maximize reporter dynamic range while ensuring compound hits reflected effects on Mtb Cl^−^ response (Fig 1B) [9]. A total of 50,816 compounds from The Rockefeller University’s High-Throughput and Spectroscopy Center were screened, with a robust average Z′-factor of 0.71 ± 0.05. The threshold for calling hits was set at 3 standard deviations from the mean and resulted in 69 hits that increased reporter induction and 67 hits that decreased reporter induction as compared to DMSO-treated control samples. As inhibition of Mtb growth confounds analysis of *rv2390c′*::GFP reporter induction, 43/67 “hits” that decreased reporter signal upon exposure to high [Cl^−^] but also exhibited significant inhibition of bacterial growth were excluded from further follow-up.

As a first validation of the results from the primary screen, the *rv2390c′*::GFP assay was repeated with a dose response curve for all called hits in triplicate. In accord with the robustness of the screen, the majority of hits were validated, with 68/69 compounds that increased reporter induction and 21/24 compounds that decreased reporter induction exhibiting the phenotype observed in the primary screen (Fig 1C). These validated compounds were then used in assays with a second, independent, Cl^−^/pH-responsive reporter, *rv1405c′*::GFP, to focus on compounds that more generally affect Mtb Cl^−^ response versus those specific for effects on *rv2390c* expression. In this secondary screen, 49/68 compounds that increased *rv2390c′*::GFP reporter induction upon exposure to high [Cl^−^], and 18/21 compounds that decreased *rv2390c′*::GFP reporter induction, also showed similar phenotypes with the *rv1405c′*::GFP reporter (Fig 1D and S1 Table).

Liquid chromatography–mass spectrometry (LC–MS) carried out on 32 resupplied hit compounds (16 that increased reporter induction and 16 that decreased reporter induction) was utilized to validate compound identity and purity. In all cases, the observed parent mass was consistent with the hit chemical structure. The chromatograms demonstrated 13/16 compounds in each class showed >99% purity, and no compound exhibited less than 88% purity.

### Small subset of compounds identified inhibit Mtb growth in host macrophages

With these hit compounds identified, we next conducted a tertiary screen to examine the effects of the selected compounds on the ability of Mtb to grow in host macrophages and to aid in prioritization of compounds for follow-up study. J774 murine macrophage-like cells were seeded in 384-well plates, infected with Mtb constitutively expressing the fluorophore monomeric Kusabira Orange (mKO) (S1 Fig), and treated with compounds in a dose–response curve. Mtb colonization of the host cells was tracked by analyzing mKO fluorescence 6 days post-infection and compared to DMSO-treated samples or cells treated with 5 μM rifampicin, a first-line Mtb drug. With the 5 μM rifampicin samples set as 100% inhibition of Mtb growth, 11 compounds were found to inhibit Mtb growth in J774 cells >20% at 20 μM (S2 Table). Among these 11 compounds, there were 2 pairs of compounds in which the paired structures were closely related analogs (Fig 1E), and where the phenotypes observed closely matched between the paired compounds. In each case, *rv2390c′*::GFP reporter signal was increased upon Mtb exposure to high [Cl^−^] and in the presence of compound treatment, and bacterial growth in J774 host cells was inhibited approximately 50% at 20 μM compound (Fig 1F). Based on the strength of these phenotypes, 1 compound from each of these pairs (termed “C5” and “C6”) was selected for continued follow-up.

Commercially purchased C5 and C6 reproduced the increased *rv2390c′*::GFP reporter signal phenotype upon Mtb exposure to high [Cl^−^], as well as the phenotype of Mtb growth inhibition during J774 infection (Fig 1G and 1H). Further, treatment of primary murine bone marrow–derived macrophages (BMDMs) with 10 μM C5 or C6 resulted in a significant decrease in bacterial load observed versus DMSO mock-treated samples in an 8-day infection time course (Fig 1I).

Together, these results identified C5 and C6 as compounds that perturb Mtb response to environmental Cl^−^ and that inhibit Mtb growth in host macrophages.

### Compound C6 exhibits promising drug-like properties

Given the marked ability of C5 and C6 to inhibit Mtb growth in host macrophages, we pursued examination of their physiochemical and PK properties with a long-term goal of assessing their potential for in vivo efficacy studies. Both compounds exhibited acceptable [27] kinetic aqueous solubility (S) in pH 7.4 phosphate buffered saline (PBS) with values of 6.67 μM (C5) and 76.4 μM (C6). Stability, as judged by half-life (t_1/2_) in the presence of mouse liver microsomes, was assessed for each compound to be below the target value of 60 minutes [27]. The C6 t_1/2_ value of 48.5 minutes was superior to that for C5 (t_1/2_ = 4.98 minutes). The greater solubility and metabolic stability of C6 as compared to C5 were reflected in its higher plasma exposure observed in CD-1 mice (*n =* 2) post a single 5 mg/kg oral (po) dose. Specifically, C6 exhibited greater area under the curve over 5 hours (AUC_0–5h_ = 5,481 h*ng/ml) than C5 (AUC_0–5h_ = 42 h*ng/ml).

With the superior PK profile at 5 mg/kg, C6 was further profiled, beginning with the determination of its plasma AUC_0–5h_ to be 25,713 h*ng/ml with a single 25 mg/kg po dose. Its 5-hour partitioning between plasma and lung was characterized by a C_lung_/C_plasma_ = 0.86. C6 was shown to lack significant toxicity (CC_50_ > 50 μg/ml; where CC_50_ = minimum compound concentration to inhibit cell growth by 50%) to in vitro cultured Vero cells, a widely utilized model mammalian cell line for compound toxicity assays [28]. C6 exhibited a slightly shorter t_1/2_ (37.6 minutes) in the presence of human liver microsomes as compared to mouse liver microsomes. The compound was characterized by high, but not prohibitively large, protein binding to mouse (97.0%) or human (99.2%) plasma. C6 exhibited sufficient stability in the presence of mouse (>99.9% remaining) or human (94.6% remaining) plasma after 5-h incubation. Inhibition of human CYP 450 enzymes was negligible, with 10 μM C6 affording <1, 25.7, 30.3, 10.5, and <1% inhibition of the 1A2, 2C9, 2C19, 2D6, and 3A4/5 isoforms, respectively. Finally, hERG inhibition was quantified by an IC_50_ = 1.2 ± 0.1 μM. Reduction of hERG inhibition, along with enhancements in mouse liver microsome stability and mouse oral exposure, will thus be goals in future analogs for this chemical series.

### Structure–activity relationship analysis reveals that the chloride response phenotype of C6 tracks with inhibition of Mtb growth in macrophages

**Fig 2:**
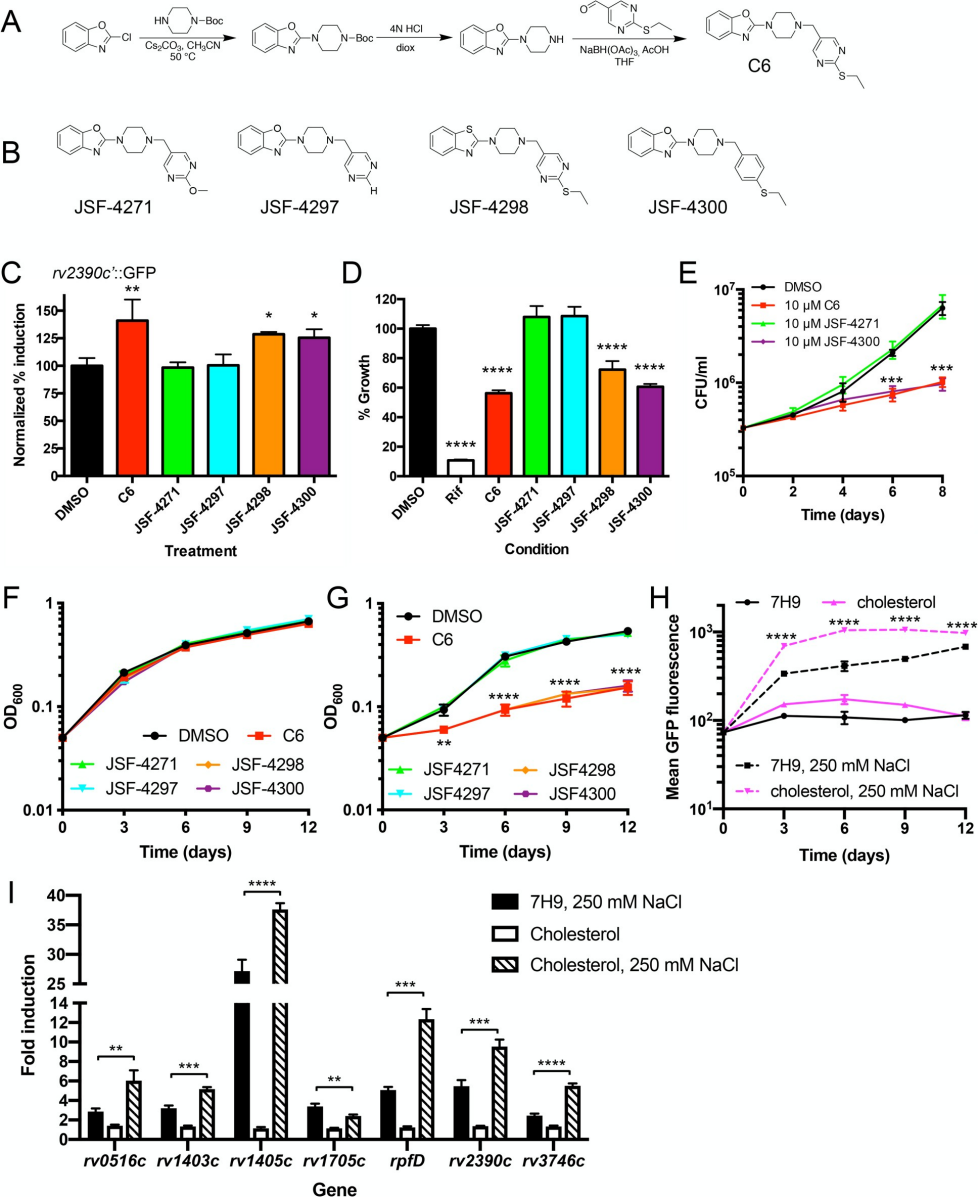
SAR analysis reveals that the chloride response phenotype of C6 tracks with inhibition of Mtb growth in macrophages. (A) Schematic for synthesis of C6. (B) Structures of C6 analogs synthesized. (C–E) C6 analogs that lose the ability to increase Mtb response to high [Cl^−^] also lose the Mtb growth inhibition phenotype in J774 cells and primary BMDMs. (C) The *rv2390c′*::GFP reporter Mtb strain was grown in 7H9 (pH 7) ± 250 mM NaCl and treated with DMSO, 10 μM C6, or 10 μM C6 analogs (JSF-4271, JSF-4297, JSF-4298, or JSF-4300) for 9 days and samples fixed and reporter GFP induction analyzed by flow cytometry. Data are shown as means ± SD from 3 independent experiments. *p*-values were obtained with a one-way ANOVA with a Dunnett multiple corrections test, and treatment sets compared to the DMSO control. *p < 0.05, **p < 0.01. (D) J774 macrophage-like cells were infected with Mtb constitutively expressing mKO and treated with DMSO, 5 μM rifampicin, 10 μM C6, or 10 μM C6 analogs (JSF-4271, JSF-4297, JSF-4298, or JSF-4300). Bacterial growth was tracked by fluorescence, with readings taken 6 days post-infection. DMSO is the carrier control, and growth in that condition is set at 100%. Data are shown as means ± SD from 4 wells. *p*-values were obtained with a one-way ANOVA with a Dunnett multiple corrections test, and treatment sets compared to the DMSO control. ****p < 0.0001. (E) BMDMs were infected with WT Mtb and bacterial load determined at indicated times. DMSO as a carrier control, 10 μM C6 or 10 μM C6, or 10 μM C6 analogs (JSF-4271, JSF-4297, JSF-4298, or JSF-4300) was added 2 hours post-infection. Data are shown as means ± SD from 3 wells. *p*-values were obtained with an unpaired *t* test, comparing each treatment to the DMSO control for a given time point, and apply to both C6 and JSF-4300. ***p < 0.001. (F) C6 does not affect Mtb growth in standard 7H9 media. Mtb was grown in standard 7H9 media and cultures treated with DMSO or 10 μM of indicated compounds. OD_600_ was tracked over time. Data are shown as means ± SD from 3 independent experiments. (G) C6 analogs that lose the ability to increase Mtb response to high [Cl^−^] also lose the Mtb growth inhibition phenotype in cholesterol media. Mtb was grown in cholesterol media and cultures treated with DMSO or 10 μM of indicated compounds. OD_600_ was tracked over time. Data are shown as means ± SD from 3 independent experiments. *p*-values were obtained with an unpaired *t* test, comparing each treatment to the DMSO control for a given time point, and apply to C6, JSF-4298, and JSF-4300. **p < 0.01, ****p < 0.0001. (H) High [Cl^−^] in the presence of cholesterol media increases expression of the *rv2390c′*::GFP reporter. The *rv2390c′*::GFP reporter Mtb strain was grown in 7H9 (pH 7) ± 250 mM NaCl or cholesterol media (pH 7) ± 250 mM NaCl, and samples fixed every 3 days for 12 days. Reporter GFP signal was analyzed by flow cytometry, and data are shown as means ± SD from 3 independent experiments. *p*-values were obtained with an unpaired *t* test, comparing the high [Cl^−^]/cholesterol to the high [Cl^−^] condition for a given time point. ****p < 0.0001. (I) Mtb response to high [Cl^−^] is augmented in the presence of cholesterol. qRT-PCR of gene expression of WT Mtb exposed to 7H9 (pH 7) ± 250 mM NaCl or cholesterol media (pH 7) ± 250 mM NaCl, for 4 hours. Data are shown as means ± SD from 3 technical replicates, representative of 3 independent experiments. *p*-values were obtained with an unpaired *t* test, comparing the cholesterol, 250 mM NaCl condition to the 7H9, 250 mM NaCl condition for each gene. **p < 0.01, ***p < 0.001, ****p < 0.0001. The numerical data underlying the graphs shown in this figure are provided in S1 Data. BMDM, bone marrow–derived macrophage; mKO, monomeric Kusabira Orange; Mtb, *Mycobacterium tuberculosis*; qRT-PCR, quantitative real-time PCR; SAR, structure–activity relationship; WT, wild-type.

To begin to probe some of the basic SAR for C6, we devised a scalable synthesis of C6 that could readily be extended to the synthesis of analogs. C6 was synthesized in 3 steps from commercially available 2-chlorobenzo[*d*]oxazole involving displacement with piperazine followed by reductive amination with commercial 2-(ethylthio)pyrimidine-5-carbaldehyde in 60.3% overall yield (Fig 2A). This route was adapted through the use of 2-methoxypyrimidine-5-carbaldehyde, pyrimidine-5-carbaldehyde, or 4-(ethylthio)benzaldehyde to prepare JSF-4271, JSF-4297, and JSF-4300, respectively (Fig 2B). 2-chlorobenzo[*d*]thiazole was substituted in the reaction scheme to afford JSF-4298 (Fig 2B).

Tests of the C6 analogs in the *rv2390c′*::GFP reporter assay to determine compound modulation of Mtb response to Cl^−^ showed that analogs JSF-4271 and JSF-4297 had lost the ability demonstrated by the parental C6 compound to increase reporter GFP signal in the presence of high [Cl^−^] (Fig 2C). Loss of this phenotype was also accompanied by loss of the ability to inhibit Mtb growth in J774 cells and primary murine BMDMs (Fig 2D and 2E). While C6 and its analogs have no effect on Mtb growth in standard 7H9 media (Fig 2F), we additionally examined the effect of the analogs on Mtb growth in cholesterol media, as C6 was also part of a previous independent screen for compounds that inhibited Mtb growth in cholesterol media (PubChem AID 1259343) and had been called as positive for growth inhibition in that screen (PubChem SID 340463129). In addition, V-58, a compound with structural similarity to C6, had also been reported to inhibit Mtb growth in cholesterol [29]. In agreement with the previously reported screen data, we found that C6 indeed inhibited Mtb growth in cholesterol media (Fig 2G). As with the *rv2390c′*::GFP reporter and growth in macrophage phenotypes, analogs JSF-4271 and JSF-4297 no longer inhibited Mtb growth in cholesterol media (Fig 2G).

The impact of C6 on Mtb growth in cholesterol media but not standard 7H9 media, combined with its effect on induction of the *rv2390c′*::GFP reporter in high [Cl^−^] media, led us to analyze the activity of the *rv2390c′*::GFP reporter in untreated Mtb grown in cholesterol media. Cholesterol media alone had little effect on expression of the *rv2390c′*::GFP reporter (Fig 2H), in accord with *rv2390c* not previously being reported to be part of the cholesterol regulon [30]. Intriguingly, however, we found that induction of the *rv2390c′*::GFP reporter in the presence of high [Cl^−^] was increased in the context of cholesterol media (Fig 2H). Quantitative real-time PCR (qRT-PCR) analysis of several other genes in the Cl^−^ regulon showed a similar trend of increased induction in the dual high [Cl^−^]/cholesterol media condition versus high [Cl^−^] alone for 5 of the 6 other genes tested (Fig 2I), supporting a broader relationship between Cl^−^ and cholesterol response, versus one restricted to *rv2390c*.

Together, these results reveal a potential intersection between Mtb response to Cl^−^ with its metabolism and indicate the importance of the 2-ethylthio group, with analogs JSF-4298 and JSF-4300, which retain the 2-ethylthio group, both continuing to demonstrate the phenotypes seen with the parental C6 compound, in contrast to analogs JSF-4271 and JSF-4297.

### C6 does not alter phagosome maturation characteristics

We next sought to determine if C6 exerted effects on the host cell, specifically on phagosome maturation characteristics, independent of infection. Carboxyfluorescein-linked silica beads enable ratiometric assessment of changes in macrophage phagosomal pH, via measurement of carboxyfluorescein fluorescence at pH-dependent versus pH-independent wavelengths [31–35]. As shown in S2A Fig, addition of C6 did not alter macrophage phagosomal acidification. C6 also did not affect macrophage phagosomal proteolytic activity, as demonstrated using DQ-BSA/Alexa Fluor 594-linked silica beads (S2B Fig) [31–33]. Finally, utilization of 10,10′-bis[3-carboxylpropyl]-9,9′-biacridinium (BAC)/Alexa Fluor 594-linked silica beads, reporting specifically on changes in Cl^−^ [9,36], demonstrated no significant effect of C6 on the increase in [Cl^−^] that occurs during macrophage phagosomal maturation (S2C Fig). Together, these data demonstrate that C6 does not affect macrophage phagosomal maturation characteristics independent of infection.

### C6 accumulates in Mtb and host macrophages

**Fig 3:**
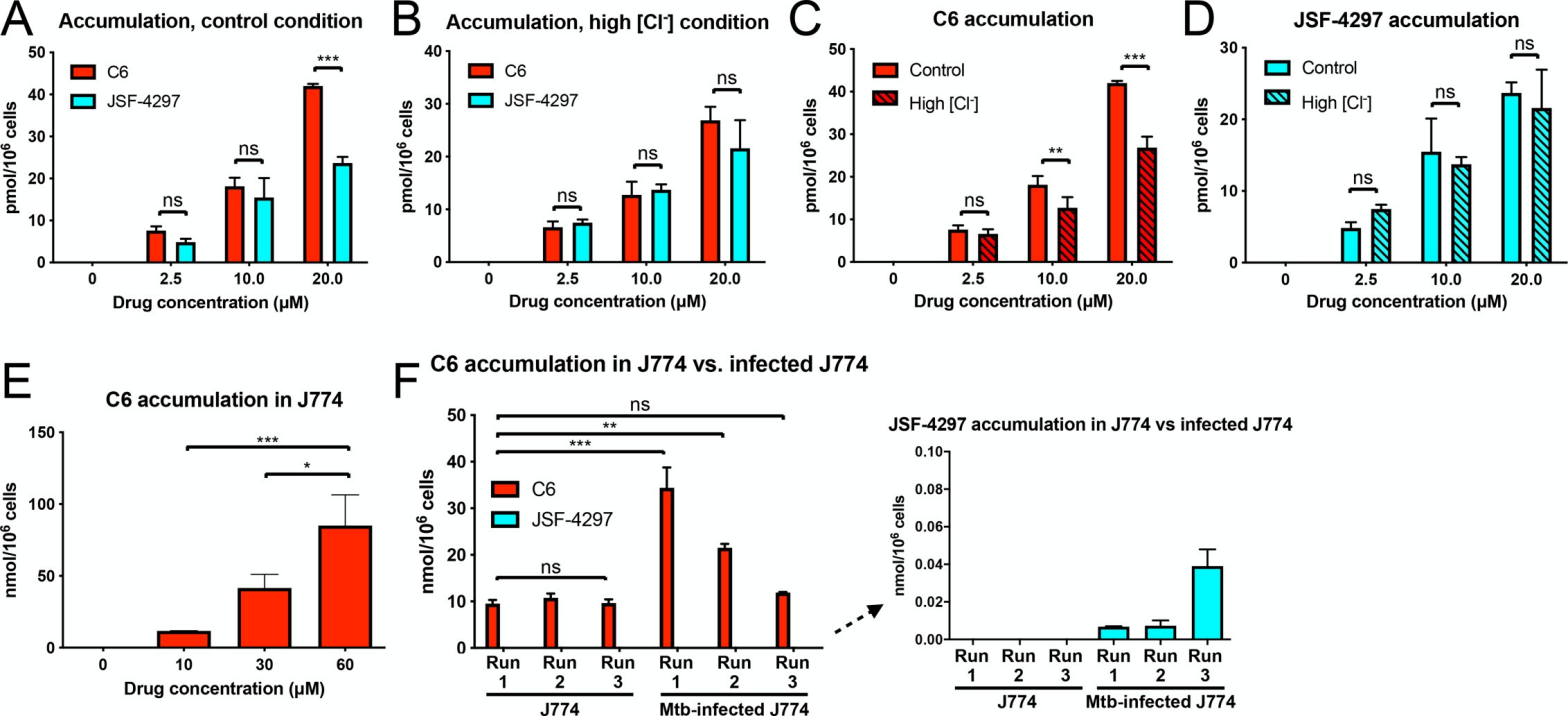
C6 accumulates in Mtb and host macrophages. (A–D) Intrabacterial accumulation of C6 is higher than the inactive analog JSF-4297. Mtb were grown in 7H9 (pH 7) ± 250 mM NaCl for 6 days, before 24-hour exposure to C6 or JSF-4297 and analysis of intrabacterial compound content. (A) and (B) show dose-dependent intrabacterial accumulation of C6 versus JSF-4297 under control and high [Cl^−^] conditions, respectively. (C) and (D) show the same data but compare C6 or JSF-4297 accumulation under control versus high [Cl^−^] conditions in each case. (E) Intracellular accumulation of C6. J774 cells were exposed to indicated concentrations of C6 for 24 hours, before analysis of the samples for intracellular compound content. (F) C6 accumulation during Mtb infection of J774 cells. J774 cells were infected with Mtb for 5 days, before treatment with 10 μM of indicated compound for 24 hours, sample collection and analysis for total compound accumulation (within both J774 cells and bacteria within the J774 host cells). For (A–E), data are shown as means ± SD from 2–3 samples, representative of 2 independent experiments. In (F), data are shown as means ± SD from 2–3 wells, from 3 independent experiments. *p*-values were determined by one- (E) or two-way (A–D, F) ANOVA with Bonferroni post hoc test for all assays. ns *p* > 0.05, *p < 0.05, **p < 0.01, ***p < 0.001. The amount of accumulated compound as the number of moles was normalized by the cell number (Mtb or J774) prior to compound incubation. The numerical data underlying the graphs shown in this figure are provided in S1 Data. Mtb, *Mycobacterium tuberculosis*; ns, not significant.

Since we observed the modulation of response to high [Cl^−^] in Mtb cells and inhibition of Mtb growth inside J774 macrophage-like cells by C6, we sought to confirm if these observed effects correlated with C6 accumulation in the Mtb and J774 cells. To quantify the intrabacterial accumulation of C6, we leveraged our recently developed label-free cell-based methodology that enables monitoring of drug accumulation/metabolism inside bacterial cells [37,38]. This methodology generally features a cold acetonitrile/methanol/water quench of metabolism and extraction of cellular metabolites and downstream identification and quantification of drug and drug metabolites via LC–MS [37,38]. Utilizing this platform, we measured C6 accumulation in Mtb after 24-hour incubation of the bacteria under control or high [Cl^−^] media conditions, in the presence of a range of C6 concentrations (0 to 20 μM). This assay revealed dose-dependent C6 accumulation in the bacteria (Fig 3A–3C). Experiments with the inactive analog JSF-4297 showed that while this compound accumulated inside Mtb cells, it did so at levels lower than that observed with C6 and with no statistically significant difference in accumulation in the control versus high [Cl^−^] media conditions (Fig 3A, 3B and 3D).

We then explored whether C6 could accumulate in the host J774 cells. With a modified protocol, we incubated J774 cells with 0 to 60 μM C6 for 24 hours. A dose-dependent accumulation of C6 in J774 cells, similar to that observed in Mtb, was observed (Fig 3E). We further confirmed C6 accumulation in the Mtb-infected J774 cells after incubating the cells with 10 μM C6 for 24 hours (Fig 3F), extending our scope to systems with bacteria-infected eukaryotic cells. There was a trend of greater C6 accumulation in the Mtb-infected J774 cells than uninfected J774 cells, although significant variation in accumulation was seen among experimental runs with the infected J774 cells (Fig 3F). In striking contrast, we observed no detectable accumulation when we incubated J774 cells with JSF-4297 (Fig 3F). In accord with these results, analyses of compound accumulation upon treatment with JSF-4271 (inactive analog) or JSF-4300 (active analog) showed decreased accumulation of JSF-4271 in Mtb, and minimal accumulation of JSF-4271 in host J774 cells or in samples extracted from Mtb-infected J774 cells, while robust accumulation was observed with the active analog JSF-4300 (S3 Fig). These results thus suggest an interesting and unexpected tie between compound structure and accumulation in bacterial and J774 host cells.

### C6 inhibits Mtb growth *in vivo*

**Fig 4:**
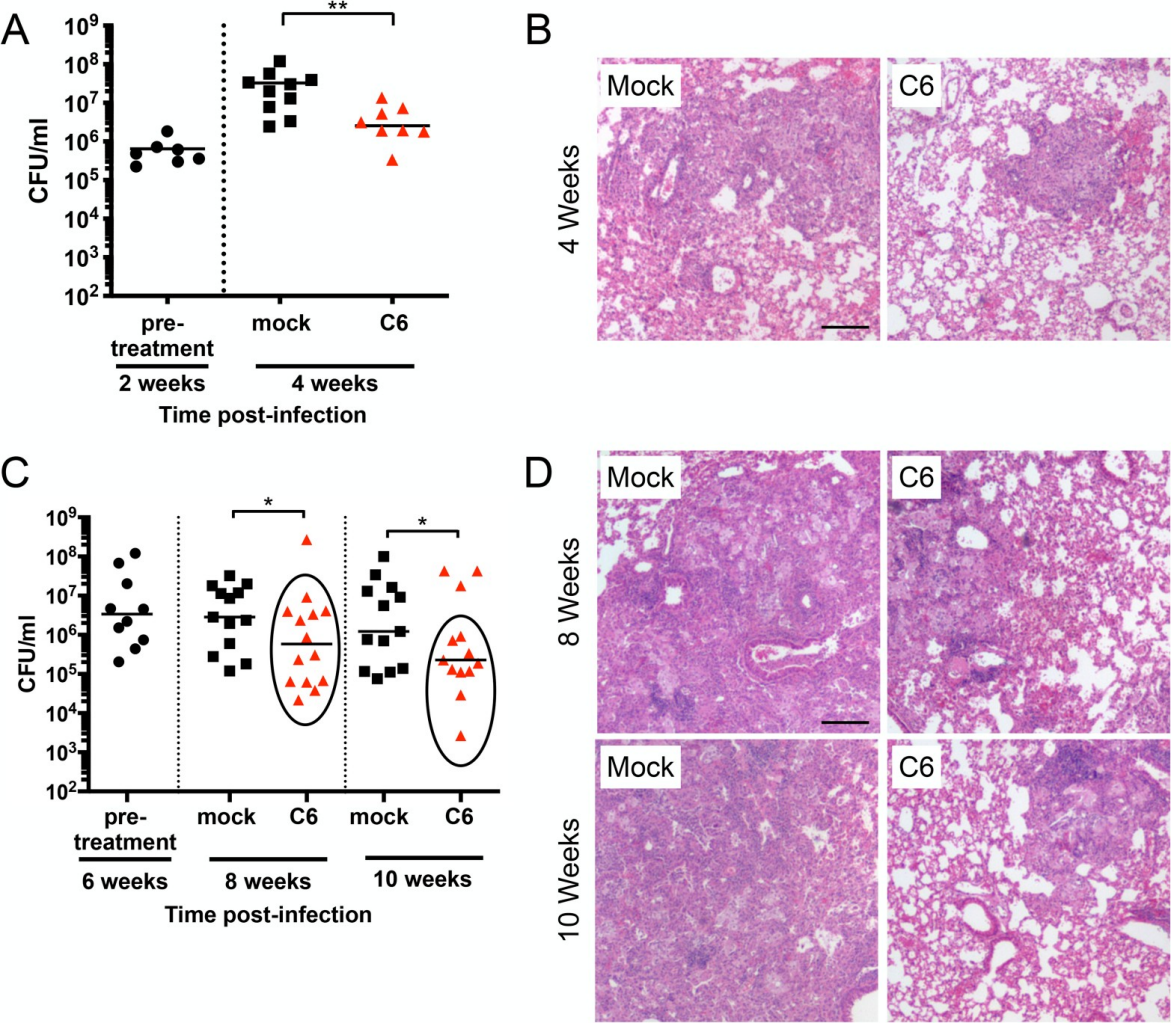
C6 inhibits Mtb growth in vivo. (A and B) C6 inhibits Mtb growth in a short-term infection model. C3HeB/FeJ WT mice were infected with Mtb for 2 weeks (pre-treatment), before mock treatment or treatment with 250 mg/kg C6 (5 days/week via oral gavage) for a further 2 weeks. (A) shows CFUs from lung homogenates plated at 2 or 4 weeks post-infection. *p*-values were obtained with a Mann–Whitney statistical test. **p < 0.01. (B) shows lung pathology in the infected mice, with lung samples obtained from animals 2 weeks or 4 weeks post-infection, fixed, and processed for hematoxylin and eosin staining. Scale bar, 200 μm. (C and D) C6 decreases Mtb load in a longer-term infection model. C3HeB/FeJ WT mice were infected with Mtb and infection allowed to establish for 6 weeks. Mice were then mock treated or treated with 250 mg/kg C6 by oral gavage 5 days/week for 2 or 4 weeks (8 or 10 weeks total infection). Lungs were homogenized and plated for CFUs at indicated time points, shown in (C). *p*-values were determined by a Mann–Whitney statistical test on the mock versus circled C6-treated population. *p < 0.05. (D) shows lung pathology in infected mice, with lung samples obtained from animals 6, 8, or 10 weeks post-infection, fixed, and processed for hematoxylin and eosin staining. Scale bar, 200 μm. The numerical data underlying the graphs shown in this figure are provided in S1 Data. CFU, colony-forming unit; Mtb, *Mycobacterium tuberculosis*; WT, wild-type.

Given the promising results above of C6 inhibition of Mtb growth in host macrophages and its favorable PK profile, we finally sought to assess if C6 had in vivo efficacy. For these experiments, we utilized the C3HeB/FeJ murine infection model, as Mtb infection in these mice recapitulates key lesion types observed during human infection, and its use in studies of compound effects on Mtb treatment has thus become increasingly appreciated [3,39–42]. To begin, we tested the effects of C6 upon short-term Mtb infection, where treatment was initiated prior to the onset of adaptive immunity. Specifically, we infected C3HeB/FeJ mice with Mtb and allowed the infection to establish for 2 weeks. We then began treatment with 250 mg/kg C6 or vehicle (0.5% carboxymethyl cellulose (CMC) + 0.5% Tween 80) for a further 2 weeks, via oral gavage 5 times a week. Lungs were harvested 2 or 4 weeks post-infection and the bacterial load quantified. Excitingly, this early 2-week treatment with C6 significantly inhibited Mtb growth in the mice lungs, with histological examination showing correspondingly less cellular infiltration (Fig 4A and 4B).

To extend on this result, we next pursued tests of the effect of C6 on a longer-term infection model in C3HeB/FeJ mice, to determine if inhibition of Mtb growth would still be observed post-formation of caseous necrotic lesions, a hallmark lesion type observed during human infection [42]. This is of specific interest as the effects of antitubercular drugs have been shown to differ depending on whether caseous necrotic lesions are formed [3,5,39,41,42]. For example, pyrazinamide and bedaquiline demonstrate uniform efficacy across animals in BALB/c mice, which do not form caseous necrotic lesions [5,39]. In contrast, these 2 drugs demonstrate a heterogeneous response in C3HeB/FeJ mice, with a subset of mice failing to respond to treatment [5,39]. In C3HeB/FeJ mice, caseous necrotic lesions can be observed 6 weeks post-infection with Mtb. We thus infected mice with Mtb and allowed the infection to establish for 6 weeks, before treating with 250 mg/kg C6 or vehicle for 2 or 4 weeks. Strikingly, C6 treatment resulted in a significant decrease in bacterial load at both the treatment time points, with corresponding improved histopathology (2 or 4 weeks of treatment, 8 or 10 weeks of total infection time) (Fig 4C and 4D). Of note, a few outlier mice where bacterial load remained high were present within the C6-treated population (Fig 4C). This is in accord with results previously observed with pyrazinamide and bedaquiline [5,39] and likely reflects the heterogeneity of the infection observed in C3HeB/FeJ mice. Further studies will be needed to elucidate the exact reasons for the failure of a subset of mice to respond to C6 treatment.

Together, these experiments demonstrate the ability of C6 to exert in vivo efficacy in a murine model that recapitulates key lesion types observed during human infection, significantly decreasing bacterial load and improving pathology.

## Discussion

Successful colonization of the host by Mtb requires proper sensing and response to environmental cues for bacterial adaptation. In screening for compounds that perturb the response of Mtb to Cl^−^, we have uncovered a compound, C6, which inhibits Mtb host colonization in vivo. Our study thus illuminates how examination of Mtb response to an abundant ion like Cl^−^, an understudied facet of Mtb–host interactions that nonetheless has clear connections to other critical environmental cues and relevance to Mtb infection biology [9,16], can be exploited in the discovery of compounds that disrupt Mtb response to environmental cues and host colonization.

Our SAR studies have begun to shed light on the structural components important for the phenotypes observed and illustrate the correlation between perturbation of Cl^−^ response and inhibition of Mtb growth in host cells in a subset of compounds. The inhibition of Mtb growth in cholesterol by C6 suggests complexity in its mechanism of action that may extend beyond modulation of Mtb response to environmental cues. Of note, however, a second compound with shared structural components to C6 (PubChem SID: 340463409) that was also called as having antitubercular activity in cholesterol media in that assay set (PubChem AID 1259343) did not demonstrate dysregulation of Mtb Cl^−^ response in our original *rv2390c′*::GFP screen. This is in accord with our SAR results, as it lacks the 2-ethylthio group essential to date for our Cl^−^ response phenotype within this general class of compounds and indicates that the structural components that drive the Cl^−^ response phenotype versus the cholesterol phenotype can be separated. Further studies will be required to tease this aspect apart and to delineate the relative contribution of the impact of C6 on Mtb Cl^−^ response dysregulation versus cholesterol utilization on the in vivo bacterial growth inhibition phenotype. This is of particular interest as the compound screens conducted here also revealed compounds that dysregulate Mtb Cl^−^ response but did not affect growth of the bacteria in J774 macrophage-like cells. These additional studies will thus be important in elucidating whether Cl^−^ response dysregulation can drive a compound’s in vivo antitubercular activity, versus act in a secondary manner, with a different mechanism of action as the primary driver. The simultaneous presence of both high [Cl^−^] and cholesterol in the local environment is anticipated during the course of Mtb infection, for example, in the context of Mtb residing in foamy macrophages. Together with our unexpected finding that induction of the *rv2390c′*::GFP reporter in response to high [Cl^−^], and of several other genes in the Cl^−^ regulon, is increased in the context of cholesterol media, these results suggest an intriguing potential intersection between Mtb Cl^−^ response and its metabolism, and we anticipate that further work on this facet will reveal new insight into how Mtb coordinates environmental response to its metabolism.

We have additionally noted the structural similarity of C6 with compound V-58, which also inhibits Mtb growth in cholesterol, with the pyrimidine 5-position in both compounds linked by a methylene to a heterocycle (piperazine 4-position in C6 and piperidine 4-position for V-58) [29]. The remaining nitrogen of the piperazine in C6 and piperidine in V-58 are both attached to a nitrogen-containing heterocycle [29]. V-58 has been elegantly demonstrated to activate Rv1625c and thus promote cAMP synthesis [29]. While we have not initiated a further comparison of both compounds, we expect that future studies will delineate any mechanistic overlap between dysregulation of Mtb response to high [Cl^−^], activation of Rv1625c, and modulation of cAMP synthesis.

Reporter Mtb strains are unique and powerful tools that have provided insight into various aspects of Mtb biology in vitro and in vivo [7,9,16,19,22–26], and our study reinforces their utility in uncovering compounds that can serve as chemical probes for understanding Mtb environmental cue response, and with the potential for development as lead compounds. For example, the increase in *rv2390c′*::GFP reporter signal observed in high [Cl^−^] conditions upon Mtb treatment with C6 suggests a possibility that C6 may act to inhibit a transcriptional repressor involved in Mtb Cl^−^ response. We anticipate that chemical/genetic epistasis experiments exploiting the use of C6 in combination with Mtb mutants will thus add to our knowledge of the underlying network regulating Mtb Cl^−^ response. Additionally, analyses of the intrabacterial and intracellular accumulation of compounds represent approaches with broad applicability to study the general phenomenon of drug/chemical probe interactions within the cell. As shown here, they enable analysis of compound accumulation within the bacterium and/or host cells, independently or in the combined context of infection, revealing how differential accumulation may contribute to differences in compound antibacterial activity. These approaches are complementary to examples of intrabacterial drug metabolism from our lab and others [37,38,43–45].

Particularly given the growing understanding of the intimate connections between homeostasis and response to disparate ionic cues [16], and the intersection of Mtb environmental response with its metabolism [46], we propose that this facet of Mtb–host interactions represents an area with meaningful potential in the development of new antituberculars, which may also exhibit synergy with current drugs. In addition, chemical probes identified in screens such as these provide new tools that can be exploited in further understanding the molecular mechanisms underlying Mtb response to a given environmental cue, providing critical insight into fundamental aspects of Mtb biology.

## Materials and methods

### Ethics statement

Animal protocols were reviewed and approved by the Institutional Animal Care and Use Committee at Tufts University (#B2019-10), or the Center for Discovery and Innovation, Hackensack Meridian Health (#269.01), in accordance with the Association for Assessment and Accreditation of Laboratory Animal Care, the US Department of Agriculture, and the US Public Health Service guidelines. All animal procedures adhered to the National Institutes of Health “Guide for the Care and Use of Laboratory Animals” standards.

### Mtb strains and culture

Mtb strains for bacterial growth and growth inhibition assays with J774 cells were in the CDC1551 background. The Mtb strains for primary murine BMDM infections and mouse infections were in the Erdman background. CDC1551(*rv2390c′*::GFP) was used for the chemical compound screen and has been previously described [9]. CDC1551(*rv1405c′*::GFP) was generated in a similar manner, with a 744-bp region immediately upstream of the *rv1405c* open reading frame PCR amplified and cloned in front of GFPmut2 in the modified replicating plasmid pSE100, and the resultant plasmid transformed into Mtb. To generate the P1′::mKO construct, a codon-optimized mKO driven by the P1 promoter was introduced into the pDE43-MEK vector by Gateway cloning [47,48]. The reporter was then transformed into CDC1551, with selection on 7H10 agar containing 25 μg/ml kanamycin. Routine propagation of Mtb cultures were as previously described, in standing T25 flasks with filter caps, in 7H9 Middlebrook medium supplemented with OADC, 0.05% Tween 80, buffered at pH 7.0 with 100 mM MOPS [19]. A quantity of 50 μg/ml hygromycin B or 25 μg/ml kanamycin was added as needed for maintenance of reporter plasmids. Preparation of Mtb stocks used for mice infections was as previously described [22].

### Small molecule chemical screen with fluorescent reporter Mtb strain

The CDC1551(*rv2390c′*::GFP) reporter Mtb strain was utilized for the small molecule compound screen. Screening compounds were from The Rockefeller University’s High-Throughput and Spectroscopy Center and were aliquoted at 20 μM in 25 μl 7H9 (pH 6.4), 250 mM NaCl media in bar-coded 384-well black, clear-bottom plates. Mid-log phase (OD_600_ approximately 0.6) reporter Mtb grown in standard 7H9 (pH 7) media were resuspended in 7H9 (pH 6.4), 250 mM NaCl media, and 25 μl of the bacterial suspension added to each well, at a final OD_600_ = 0.05 (final compound concentration = 10 μM), using a Janus Ministation (PerkinElmer, Waltham, MA). For each test plate, 2 columns of controls (16 wells each) containing DMSO as a carrier control were included, with 1 column consisting of Mtb in 7H9 (pH 6.4) media (negative control), and the other column consisting of Mtb in 7H9 (pH 6.4), 250 mM NaCl media (positive control). Bacteria in all control wells were also inoculated at a final OD_600_ = 0.05. All wells (test and control) also contained 50 μg/ml hygromycin B and 100 μg/ml cycloheximide. Plates were kept in humidified ziplock bags in a 37°C, 5% CO_2_ incubator. GFP signal (top read monochromator, excitation 488 nm, emission 510 nm) and OD_600_ were measured 6 days post-inoculation with a PerkinElmer EnVision multimode plate reader.

Validation tests of called hits and assays with the *rv1405c′*::GFP reporter Mtb strain were done as dose–response assays in triplicate runs, with compounds tested beginning at a final concentration of 20 μM and 2-fold dilutions down to 0.039 μM.

### Liquid chromatography–mass spectrometry authentication of screen compounds

Compounds were dissolved in HPLC-grade DMSO to 5 mM and diluted 1:25 in HPLC-grade methanol. Samples (2 μl volumes) were injected via the autosampler of an Agilent Infinity 1260 HPLC system, set to a flow rate of 200 μl/minute. A gradient of 5% to 90% acetonitrile in 0.1% formic acid was developed over 6 minutes on a ZORBAX Rapid Resolution HTExtend 1.8 μm C18 80 Å column (2.1 × 50 mm), with a 600-bar pressure limit. The column eluant was analyzed using an Agilent 6230 TOF Mass Spectrometer/PDA detector, and purity was determined by AUC of total ion current and absorbance at 208 nm wavelength. Caffeine and DMSO alone were used as positive and negative controls, respectively.

### J774 cell culture and infection

For the tertiary screen of selected hit compounds in J774 murine macrophage-like cells, 30,000 J774 cells/well were seeded in bar-coded 384-well black, clear bottom plates in a 30-μl volume in J774 infection media (DMEM + 10% FBS + 1 mM sodium pyruvate + 2 mM L-glutamine) using a ThermoFisher Multidrop Combi reagent dispenser, 1 day prior to assay start. On the day of the assay, log-phase Mtb constitutively expressing mKO were pelleted and resuspended in 1 ml basal uptake buffer (0.5% bovine serum albumin + 25 mM dextrose + 0.5 mM MgCl_2_ + 1 mM CaCl_2_ + 0.1% gelatin in PBS, passed 6× through a tuberculin syringe with a 25 G × 5/8″ needle, then resuspended in J774 infection media to a final OD_600_ = 0.2. A volume of 10 μl of this bacterial suspension was added to each well of the 384-well plates containing the J774 cells, followed by addition of 10 μl of the appropriate compounds (prealiquoted in v-bottom plates in a 1:1 mix of PBS:J774 infection media). Addition of Mtb and compounds was conducted using a Janus Ministation. For each test plate, 2 columns of controls (16 wells each) were included, with 1 column consisting of treatment with DMSO as a carrier control (negative control) and the other column consisting of treatment with 5 μM rifampicin (positive control). This tertiary assay was done as a dose–response assay in duplicate runs, with compounds tested beginning at a final concentration of 20 μM and 2-fold dilutions down to 0.039 μM. Plates were kept in humidified ziplock bags in a 37°C, 5% CO_2_ incubator. Six days post-infection, mKO fluorescence signal was read using a PerkinElmer EnVision multimode plate reader (bottom read filter, excitation 530/8 nm, Bodipy TMR D555 single mirror, emission 579/25 nm). mKO fluorescence in DMSO-treated control samples were defined as 100% growth, and fluorescence observed in treatment conditions calculated as a percentage from that set value.

For J774 infection in 96-well plates, 1.8 × 10^5^ J774 cells were seeded per well in 180 μl of J774 infection media 1 day prior to infection. On the day of the assay, log-phase Mtb constitutively expressing mKO were pelleted and resuspended in 1 ml basal uptake buffer, passed 6× through a tuberculin syringe with a 25 G × 5/8″ needle, then resuspended in J774 infection media to a final OD_600_ = 0.2. Media was removed from 96-well plate containing the J774 cells, with 140 μl of fresh J774 infection media added back to each well. A volume of 40 μl of the Mtb suspension at OD_600_ = 0.2 was added to each well, followed by addition of appropriate concentration of compound or DMSO in a 20-μl final volume in J774 infection media (compound final concentration 10 μM, rifampicin final concentration 5 μM, DMSO final concentration 0.1%/well). Bacterial mKO fluorescence was read immediately after infection using a Biotek Synergy Neo2 microplate reader (bottom read monochromator, excitation 543/10 nm, emission 565/10 nm), and again 6 days post-infection. Plates were kept in humidified ziplock bags in a 37°C, 5% CO_2_ incubator. mKO fluorescence in DMSO-treated control samples were defined as 100% growth, and fluorescence observed in treatment conditions calculated as a percentage from that set value.

### Primary murine bone marrow–derived macrophage culture and infection

C57BL/6J wild-type mice (Jackson Laboratories) were used for extraction of BMDMs. Cells were harvested and expanded in DMEM containing 10% FBS, 15% L-cell conditioned media, 2 mM L-glutamine, 1 mM sodium pyruvate, and penicillin/streptomycin as needed in a 37°C incubator in a 5% CO_2_ atmosphere. Macrophage infections were performed essentially as previously described [19], with the addition of 10 μM compound C6, or DMSO as a carrier control, to the media 2 hours after bacterial inoculation, after noninternalized Mtb had been washed away. Macrophages were lysed with water containing 0.01% sodium dodecyl sulfate and a dilution series of the samples plated on 7H10 agar for CFU determination.

### Reporter Mtb strain broth assays

Broth culture assays with the CDC1551(*rv2390c′*::GFP) reporter Mtb strain was carried out essentially as previously described, in standing filter-cap T-25 flasks in 10 ml of 7H9 or cholesterol media buffered at pH 7, with addition of 250 mM NaCl as needed [9,16]. Cholesterol medium consisted of 7H9 broth supplemented with 0.5 g/l fatty acid–free bovine serum albumin, 14.5 mM NaCl, 0.2 mM cholesterol, and 0.1% tyloxapol, buffered to pH 7.0 with 100 mM MOPS [49]. Cholesterol stocks were prepared at 100 mM, in 1:1 ethanol:tyloxapol [49–51]. In brief for the reporter assay, log-phase Mtb was used to inoculate 10 ml of fresh 7H9 or cholesterol media at pH 7, or 7H9 or cholesterol media at pH 7 supplemented with 250 mM NaCl media, at a starting OD_600_ = 0.05, with 50 μg/ml hygromycin B added to maintain reporter plasmid selection. DMSO as a carrier control or 10 μM of compound to be tested was added at the start of the assay as appropriate. Mtb was fixed at indicated time points in 4% paraformaldehyde (PFA) in PBS. Analysis of bacterial fluorescence was performed using a BD FACSCalibur, and data analyzed using FlowJo (BD Biosciences, Franklin Lakes, NJ). Initial follow-up tests of C5 and C6 utilized commercially obtained compounds from Molport, SIA (Riga, Latvia).

### Compound synthesis

Reagents and solvents for chemical reactions were purchased from Sigma-Aldrich, Acros, Alfa Aesar, Tokyo Chemical Industry (TCI), or Fisher Scientific. As necessary, reactions were performed under a nitrogen atmosphere with anhydrous solvents. Instrumentation and analytical pipelines for testing of the synthesized compounds were as previously described [37,52]. In sum, an Avance 500 MHz spectrometer from Bruker (Billerica, MA) was used for determination of NMR spectra of the synthesized compounds. Low-resolution mass spectral data were recorded on an Agilent 6120 single quadrupole LC/MS system, while high-resolution data were taken with an Agilent 6220 accurate-mass time-of-flight system. All compounds utilized in the biological assays were characterized by the expected NMR and mass spectral data and had an LC purity at 250 nm ≥95%. An Agilent 6120 single quadrupole LC/MS system was used for reverse-phase high-performance liquid chromatography (HPLC), with a reverse-phase EMD Millipore Chromolith SpeedRod RP-18e column (50 × 4.6 mm). Typically, a 10% to 100% gradient of acetonitrile/water containing 0.1% formic acid was used for sample analysis. All compounds demonstrated ≥95% purity via an HPLC UV trace at 220 nm or 250 nm, with at minimum a consistent low-resolution MS *m/z* value. Sample purification via flash chromatography was achieved with a Teledyne ISCO CombiFlash Rf+ system and a Teledyne RediSep normal phase silica gel column. TLC relied on aluminum plates coated by silica gel 60 with a F_254_ fluorescent indicator (EMD Millipore). An Agilent SD-1 preparative HPLC system was used for preparative reverse-phase HPLC, with Agilent Pursuit (10 μm, 250 × 21.2 mm) C-18 columns and a detection UV wavelength of 220 nm or 250 nm. Separation was conducted with a gradient of acetonitrile in water at a flow rate of 20 ml/min.

Representative synthesis of 2-(4-((2-(ethylthio)pyrimidin-5-yl)methyl)piperazin-1-yl)benzo[*d*]oxazole (C6): *tert-*butyl 4-(benzo[*d*]oxazol-2-yl)piperazine-1-carboxylate. *tert*-butyl piperazine-1-carboxylate (2.23 g, 12.0 mmol, 1.2 eq) and cesium carbonate (3.58 g, 11.0 mmol, 1.1 eq) were diluted in 15 ml anhydrous acetonitrile at 50°C. To the stirred reaction mixture was added 2-chlorobenzo[*d*]oxazole (1.55 g, 10.0 mmol). The reaction was stirred at 50°C for 12 hours. The reaction mixture was then cooled to room temperature (RT) and solvent removed in vacuo. The resulting solid was diluted with water and extracted with ethyl acetate (3 × 50 ml). The combined organic layers were dried over anhydrous Na_2_SO_4_, and the solvent was removed in vacuo yielding the product as a white solid (3.00 g, 98.9%): ^1^H NMR (500 MHz, CDCl_3_) 7.39 (d, J = 5.0 Hz, 1), 7.28 (s, 1), 7.19 (t, J = 7.5 Hz, 1), 7.05 (t, J = 7.5 Hz, 1), 3.70 (br s, 4), 3.57 (br s, 4), 1.49 (s, 9). Calculated for C_16_H_22_N_3_O_3_ (M+H)^+^ = 304.2; Observed 304.2. This reaction product was used in the next step without purification. 2-(piperazin-1-yl)benzo[*d*]oxazole. To a round-bottom flask containing *tert*-butyl 4-(benzo[*d*]oxazol-2-yl)piperazine-1-carboxylate (3.00 g, 9.90 mmol) was added 4 N HCl in 1,4-dioxane (20 ml) dropwise at 0°C. The reaction was stirred at RT and monitored by TLC. Upon completion, the solvent was removed in vacuo, and the resulting solid was diluted with water, and taken to approximately pH 8 with saturated sodium bicarbonate aqueous solution, and extracted with ethyl acetate (3 × 50 ml). The combined organic layers were dried over anhydrous Na_2_SO_4_, and the solvent was removed in vacuo yielding the product as an off-white solid (2.00 g, 99.4%): ^1^H NMR (DMSO-d_6_) 9.60 (br s, 1), 7.45 (d, J = 5.0 Hz, 1), 7.35 (d, J = 5.0 Hz, 1), 7.19 (t, J = 7.5 Hz, 1), 7.05 (t, J = 7.5 Hz, 1), 3.70 (t, J = 4.7 Hz, 4), 3.24 (br s, 4). Also noted, 9.4 (br s), 5.7 (s, DCM), 3.6 (m), 3.3 (s, H_2_O). Calculated for C_11_H_14_N_3_O (M + H)^+^ = 204.1; Observed 204.0. This reaction product was used in the next step without purification. The final product (C6) was prepared via mixing 2-(piperazin-1-yl)benzo[*d*]oxazole (700 mg, 3.44 mmol), 2-(ethylthio)pyrimidine-5-carbaldehyde (579 mg, 3.44 mmol), and sodium triacetoxyborohydride (1.46 g, 6.88 mmol, 2.0 eq) dissolved in anhydrous tetrahydrofuran. Glacial acetic acid (0.2 ml) was added to the stirred mixture. The reaction was monitored by TLC until starting material was consumed. Upon completion, the pH was adjusted to approximately 9 with 1 M NaOH_(aq)_ and extracted with diethyl ether (3 × 100 ml). The combined organic layers were washed with saturated aqueous brine solution, dried over anhydrous Na_2_SO_4_, and the solvent was removed in vacuo. The crude product was purified by silica gel flash column chromatography with EtOAc/hexanes to afford the product as white solid (0.750 g, 61.3%): ^1^H NMR (500 MHz, CDCl_3_) δ 8.51 (br s, 2), 7.32 (d, J = 7.9 Hz, 1), 7.25 (d, J = 7.8 Hz, 1), 7.18 (t, J = 7.6 Hz, 1), 7.03 (t, J = 7.9 Hz, 1), 3.75 (br s, 4), 3.52 (br s, 2), 3.16 (q, J = 7.3 Hz, 2), 2.61 (br s, 4), 1.41 (t, J = 7.3 Hz, 3). ^13^C NMR (125 MHz, DMSO) δ 169.9, 161.8, 158.2 (2 carbons), 148.3, 142.9, 126.3, 124.0 (2 carbons), 120.6, 115.9, 108.9, 56.0, 51.5 (2 carbons), 45.2, 24.5, 14.6. Calculated for C_18_H_21_N_5_OS (M + H)^+^ = 356.1567; Observed 356.1541.

#### 2-(4-((2-methoxypyrimidin-5-yl)methyl)piperazin-1-yl)benzo[*d*]oxazole (JSF-4271)

^1^H NMR (500 MHz, acetone-d_6_) δ 8.53 (s, 2), 7.31 (d, J = 7.9 Hz, 1), 7.26 (d, J = 7.8 Hz, 1), 7.14 (t, J = 7.6 Hz, 1), 7.01 (t, J = 7.7 Hz, 1), 3.95 (s, 3), 3.70–3.67 (m, 4), 3.57 (s, 2), 2.61–2.57 (m, 4). ^13^C NMR (125 MHz, acetone-d_6_) δ 166.4, 163.2, 160.8 (2 carbons), 149.9, 144.6, 125.6, 124.8, 121.4, 117.1, 109.6, 57.4, 55.1, 52.8 (2 carbons), 46.5 (2 carbons). Calculated for C_17_H_19_N_5_O_2_ (M + H)^+^ = 326.1638; Observed 326.1611.

#### 2-(4-(pyrimidin-5-ylmethyl)piperazin-1-yl)benzo[*d*]oxazole (JSF-4297)

^1^H NMR (500 MHz, DMSO-d_6_) δ 9.12 (s, 1), 8.77 (s, 2), 7.39 (d, J = 7.9 Hz, 1), 7.30 – 7.27 (m, 1), 7.14 (td, J = 7.6, 1.0 Hz, 1), 7.01 (td, J = 7.8, 1.2 Hz, 1), 3.61 (s, 4), 3.35 (s, 2), 2.54 – 2.51 (m, 4). ^13^C NMR (125 MHz, acetone-d_6_) δ 163.2, 158.6, 158.3 (2 carbons), 149.9, 144.6, 132.7, 124.8, 121.4, 117.1, 109.6, 58.0, 53.0 (2 carbons), 46.5 (2 carbons). Calculated for C_16_H_17_N_5_O (M + H)^+^ = 296.1533; Observed 296.1504.

#### 2-(4-((2-(ethylthio)pyrimidin-5-yl)methyl)piperazin-1-yl)benzo[*d*]thiazole (JSF-4298)

^1^H NMR (500 MHz, acetone-d_6_) δ 8.57 (s, 2), 7.70 (d, J = 7.8 Hz, 1), 7.46 (d, J = 8.1 Hz, 1), 7.28 (t, J = 7.7 Hz, 1), 7.07 (t, J = 7.6 Hz, 1), 3.66–3.63 (m, 4), 3.59 (s, 2), 3.14 (q, J = 7.3 Hz, 2), 2.64–2.61 (m, 4), 1.36 (t, J = 7.3 Hz, 3). ^13^C NMR (125 MHz, acetone-d_6_) δ 171.8, 169.3, 159.0 (2 carbons), 154.1, 132.0, 127.6, 126.9 (2 carbons), 122.3, 121.8, 119.9, 57.6, 53.0 (2 carbons), 49.3, 25.6, 15.1. Calculated for C_18_H_21_N_5_S_2_ (M + H)^+^ = 372.1338; Observed 372.1310.

#### 2-(4-(4-(ethylthio)benzyl)piperazin-1-yl)benzo[*d*]oxazole (JSF-4300)

^1^H NMR (500 MHz, acetone-d_6_) δ 7.32 (s, 4), 7.30 (s, 1), 7.26 (d, J = 7.8 Hz, 1), 7.14 (td, J = 7.7, 0.9 Hz, 1), 7.01 (td, J = 7.9, 1.1 Hz, 1), 3.69–3.66 (m, 4), 3.54 (s, 2), 2.99–2.95 (m, 2), 2.57–2.54 (m, 4), 1.28 (t, J = 7.3 Hz, 3). ^13^C NMR (125 MHz, acetone-d_6_) δ 163.3, 150.0, 144.7, 137.0, 136.4, 130.6 (2 carbons), 129.6 (2 carbons), 124.8, 121.4, 117.1, 109.6, 63.0, 53.1 (2 carbons), 46.6 (2 carbons), 27.9, 14.9. Calculated for C_20_H_23_N_3_OS (M + H)^+^ = 354.1662; Observed = 354.1634.

### Physiochemical and ADME characterization of compounds

Assays examining mouse and human liver microsomal stability, kinetic aqueous solubility, mouse and human plasma protein binding and stability, and human cytochrome P450 inhibition characteristics of the compounds were run by BioDuro (San Diego, CA), using standard protocols as previously described [37]. hERG inhibition assays were also performed by BioDuro. For this assay, HEK-293 cells stably expressing the hERG K^+^ channel were provided by the Institute of SARL (CreaCell). Cells were grown in DMEM supplemented with 10% FBS and 0.8 mg/ml G418. For electrophysiology experiments, the cells were continuously superfused by extracellular saline with 140 mM NaCl, 3.5 mM KCl, 1 mM MgCl_2_, 2 mM CaCl_2_, 10 mM dextrose, 10 mM HEPES, and 1.25 mM NaH_2_PO_4_ (pH 7.4). Compounds for the assay were dissolved in DMSO at a concentration of 10 mM for cisapride, a positive control, and 30 mM for C6, and diluted in extracellular saline to the appropriate concentration (1 nM to 1 μM for cisapride and 0.3 to 30 μM for C6). The glass micropipettes for whole-cell patch-clamp recording were filled with intracellular saline containing 20 mM KCl, 115 mM potassium L-aspartate, 1 mM MgCl_2_, 5 mM EGTA, 10 mM HEPES, and 2 mM Na_2_-ATP (pH 7.2).

The hERG current was measured at a holding potential of −80 mV and then depolarized to −50 mV for 0.5 second to test the leak current. The voltage was then depolarized to 30 mV for 2.5 seconds. The peak tail current was induced by a repolarizing pulse to −50 mV for 4 seconds. An interpulse interval of 10 seconds enabled recovery from inactivation to quantify the effect of the test article on the hERG tail current. The test compound was then added to the investigated cell from a nearby capillary, typically for another 3 minutes per concentration. The data were collected by EPC-10 amplifier and stored in PatchMaster (HEKA, Holliston, MA) software. Current amplitude values were plotted versus time to illustrate the effect of the compound and quantify the percentage of channel inhibition. The results obtained from at least 3 cells were pooled and fitted with a nonlinear regression to estimate the mean IC_50_ value.

### Pharmacokinetic assays

Two female CD-1 mice (Charles River Laboratories) were dosed once with C5 or C6 administered orally at 5 or 25 mg/kg in 5% dimethylacetamide/60% polyethylene glycol 300/35% D5W (5% dextrose in water). Blood samples were collected in K_2_EDTA-coated tubes predose, 0.5, 1, 3, and 5 hours post-dose, placed on ice, and centrifuged to recover plasma. The recovered plasma was kept at −80°C as needed prior to analysis, which was performed as previously described [37]. In brief, a Sciex Applied Biosystems Qtrap 4000 triple-quadrupole mass spectrometer coupled to an Agilent 1260 HPLC system was used for HPLC coupled to tandem mass spectrometry (LC–MS/MS) quantitative analysis of the samples, with chromatography attained using an Agilent Zorbax SB-C8 column (2.1 × 30 mm; particle size, 3.5 μm) and a reverse-phase gradient elution. The aqueous mobile phase consisted of Milli-Q deionized water with 0.1% formic acid (A), while the organic mobile phase was 0.1% formic acid in acetonitrile (B). The gradient setup was as follows: 5% to 90% B over 2 minutes, 1 minute at 90% B, then an immediate drop to 5% B and an additional 1 minute at 5% B. Quantification of molecules was carried out via multiple reaction monitoring of parent/daughter transitions in electrospray positive ionization mode, and sample analyses accepted if quality control and standard samples had concentrations within 20% of the nominal concentration. Preparation of compounds for generation of standard curves and quality control spiking solutions were performed as previously described [37]. Data were processed using Analyst software (Version 1.6.2; Applied Biosystems Sciex, Framingham, MA).

### Vero cell toxicity assay

Vero cells (African green monkey kidney epithelial cells; ATCC CCL-81) were maintained in DMEM + 10% FBS. For the cell toxicity assay, Vero cells were plated at 1 × 10^5^ cells/well in a 96-well plate and incubated for 2 to 3 hours to allow cells to settle. The test compound was dissolved in DMSO at a final concentration of 12 mg/ml. The test compound solution was then added to plated cells, resulting in final test concentrations of 50 to 0.78 μg/ml, with each concentration tested at least in duplicate. To evaluate bacterial cell viability, 20 μl of a 1:20 MTS:PMS reagent (Promega CellTiter 96 AQ_ueous_ nonradioactive cell proliferation assay kit) was added to each well after 72 hours of incubation at 37°C, and the plate then incubated for an additional 3 hours. Absorbance at 490 nm was read on a Molecular Devices SpectraMax M5 microplate reader. The CC_50_ (the minimal amount of compound that inhibited Vero cell growth by 50%) was extrapolated by plotting absorbance at 490 nm versus the concentration of untreated Vero cells in control plates.

### Mtb growth assays

Mtb grown to log-phase in 7H9 medium (pH 7.0) was used to inoculate 10 ml 7H9 medium (pH 7.0) containing 10 μM C6/C6 analogs or DMSO as a carrier control in standing, filter-cap T25 flasks, at a starting OD_600_ of 0.05. Bacterial growth was tracked by measuring OD_600_ every 3 days for 12 days. For growth assays in cholesterol medium, log-phase Mtb grown in 7H9 medium (pH 7.0) was used to inoculate 10 ml of cholesterol medium (pH 7.0), containing 10 μM C6/C6 analog, or DMSO as a carrier control, in standing, vented T25 flasks, at a starting OD_600_ of 0.05. Bacterial growth was tracked by measurement of OD_600_ every 3 days over 12 days.

### qRT-PCR analyses

Mtb grown to log-phase in 7H9 medium (pH 7.0) was used to inoculate 10 ml 7H9 (pH 7.0) ± 250 mM NaCl or cholesterol media (pH 7.0) ± 250 mM NaCl in standing, filter-cap T25 flasks, at a starting OD_600_ of 0.3. Samples were collected 4 hours post-media condition exposure, and RNA extracted as previously described [18]. qRT-PCR on cDNA synthesized from nonamplified RNA was performed as previously described [9,16,19].

### Phagosomal maturation assays

Carboxyfluorescein (pH readout), DQ-BSA/Alexa Fluor 594 (AF594) (proteolysis readout), and BAC/AF594 (Cl^−^ readout) beads were generated essentially as previously described [9,32,33,35], except that 10,10′-Bis[3-carboxylpropyl]-9,9′-biacridinium dinitrate di-NHS ester (BAC-SE) was purchased from emp Biotech LLC. BMDMs were isolated from C57BL/6J mice (The Jackson Laboratory) and maintained as described above. A quantity of 2 × 10^5^ macrophages/well were seeded into 96-well clear bottom black plates and utilized in assays 1 to 2 days post-seeding. Macrophages were first washed 3 times with prewarmed assay buffer, with fresh buffer containing 10 μM C6, or DMSO as a carrier control, then added back to each well as appropriate. Assay buffer consisted of PBS (pH 7.2), supplemented with 5% FBS, 5 mM dextrose, 1 mM CaCl_2_, 2.7 mM KCl, and 0.5 mM MgCl_2_ in the case of assays with the carboxyfluorescein or DQ-BSA/AF594 beads [31,34]. For assays with the BAC/AF594 beads, assay buffer consisted of PBS (pH 7.2), supplemented with 5% FBS, 5 mM dextrose, 1 mM calcium acetate, 1.35 mM K_2_SO_4_, and 0.5 mM MgSO_4_ [9]. Sensor beads at approximately 2 to 5 beads/macrophage in appropriate assay buffer were finally added, with data acquisition on a Biotek Synergy H1 microplate reader with bottom read signal detection started within 2 to 3 minutes of bead addition. Excitation/emission wavelengths were 450 nm/520 nm and 490 nm/520 nm for carboxyfluorescein, 490 nm/520 nm for DQ-BSA, 365 nm/505 nm for BAC, and 590 nm/617 nm for AF594. Each experiment was run with a total of 3 to 5 replicate wells/condition, with temperature maintained at 37°C throughout the assay. Wells were read every 2 minutes for 2 hours for the carboxyfluorescein bead assays, every 2 minoutes for 4 hours for the DQ-BSA/AF594 bead assays, and every 1 minute for 2 hours for the BAC/AF594 bead assays. Analyses of the results were performed as previously described [9,31,32,34].

### Intrabacterial and intracellular drug accumulation assays

Each experimental run for these assays was carried out with 2 to 3 samples. For intrabacterial drug accumulation assays, Mtb CDC1551 was grown in 10 ml of 7H9 (pH 7) medium in standing filter-cap T-25 flasks to log phase (OD_600_ approximately 0.6), and the cultures treated with various concentrations of C6 or indicated C6 analogs, or DMSO as a carrier control, for 24 hours. Samples were then pelleted, resuspended in 1 ml of prechilled extraction buffer (2:2:1 acetonitrile:methanol:water), transferred to a 2-ml screw-cap tube containing 0.5 ml of 0.1 mm zirconia/silica beads (BioSpec Products, Bartlesville, OK), and stored at −80°C prior to further processing. J774 murine macrophage-like cells were maintained in J774 infection media and seeded at 1 × 10^6^ cells/well in 6-well plates for intracellular drug accumulation assays and 2 × 10^7^ cells/flask in T-75 flasks for assays examining drug accumulation during Mtb infection of J774 cells. For intracellular drug accumulation studies, the assay was initiated 1 hour after cell seeding, by treating the cells with C6 or C6 analogs at various concentrations, or DMSO as a carrier control, for 24 hours. After the treatment period, the cells were washed 3× with cold PBS, before 1 ml of cold PBS was added to each well and the cells scraped and collected. Samples were pelleted, resuspended in 1 ml of prechilled extraction buffer, and stored at −80°C prior to further processing. For assays with infected J774 cells, infection of the J774 cells 1 day after seeding was essentially as previously described [53]. In brief, the J774 cells were infected with log-phase Mtb CDC1551 at a MOI = 5 for 2 hours, before media was removed and then replaced with fresh prewarmed media. Media was changed daily, and treatment with C6 or C6 analogs at various concentrations, or DMSO as a carrier control, initiated 5 days post-infection. After 24 hours of treatment, cells were washed twice with media and 5 ml of cold PBS added. Cells were then scraped and the samples pelleted, before resuspension in 1 ml of prechilled extraction buffer. Samples were then transferred to a 2-ml screw-cap tube containing 0.5 ml of 0.1 mm zirconia/silica beads and stored at −80°C prior to further processing.

For continued processing, samples were thawed and lysed by bead beating for the Mtb and Mtb infected J774 assays (6 × 45 seconds, 6.5 m/s, placing samples on ice for 5 minutes in between each bead beating cycle), or by 4 freeze–thaw cycles for the uninfected J774 cells (5-min freeze step in a dry ice/ethanol bath, 30-s thaw step in water, with 10-s vortexing between cycles). After lysis, the samples were centrifuged at 13,000 rpm, 10 minutes, 4°C, and the supernatant then filtered through a 0.22-μm Spin-X centrifuge tube filter (Corning Costar, Cambridge, MA) (13,000 rpm, 10 minutes, 4°C). Samples were stored at −80°C until LC–MS analysis.

For LC–MS analysis, the samples were chromatographed using a Chromolith SpeedROD column with a gradient of water and acetonitrile acidified with 0.1% formic acid, and then analyzed using an Agilent 1260 LC system coupled to an Agilent 6120 quadrupole mass spectrometer. The mass resolution ranged from 10 to 2,000 with an accuracy of ± 0.13 Da within the calibrated mass range in scan mode. Experimental compound concentrations were calculated using the method of standard addition with authentic chemical standards. Signal intensity of each experimental compound was quantified by standard curve for an authentic/independently synthesized sample and normalized by the cell number.

### Mouse Mtb infections

C3HeB/FeJ wild-type mice (The Jackson Laboratory) were infected intranasally with 10^3^ CFUs of Mtb (35 μl), under light anesthesia with 2% isoflurane [9,16,22]. Compound C6 was prepared for treatment by suspending an appropriate amount of C6 (31 to 35 mg/ml as needed for final dosage in mice at 250 mg/kg) in Ultrapure water (Invitrogen) containing 0.5% CMC and 0.5% Tween 80, and stirring the mixture overnight at 4°C. The next day, the suspension was homogenized by bead beating with 5 mm steel beads in a TOMY microtube mixer (2 × 20 seconds) and stored at 4°C until use. C6 suspension for in vivo use was used within 2 weeks of preparation. The mice were treated by oral gavage 5 times per week for 2 or 4 weeks with 250 mg/kg C6 in 0.5% CMC + 0.5% Tween 80 or mock-treated with 0.5% CMC + 0.5% Tween 80 as a vehicle control (200 μl volume). Administration began at 2 or 6 weeks post-infection as indicated. At time of sacrifice, the left lobe and accessory right lobe of the lung were homogenized in PBS + 0.05% Tween 80 and serial dilutions plated on 7H10 agar plates with 100 μg/ml cycloheximide for bacterial load determination. The remaining 3 right lobes were fixed in 4% PFA in PBS. One lobe was used for histological analysis by standard hematoxylin and eosin (H&E) staining (Tufts Comparative Pathology Services). A Nikon Eclipse E400 with a SPOT insight color digital camera was used to image the histology samples.

## Supporting information

S1 Fig mKO-expressing Mtb strain has good dynamic and linear range. Log-phase Mtb constitutively expressing mKO was resuspended at an OD_600_ = 1 in 7H9 (pH 7) and diluted in 2-fold steps. mKO fluorescence was read on a plate reader (left y-axis, orange data points), before samples serial diluted and plated on 7H10 agar plates for corresponding enumeration of CFUs (right y-axis, black data points). Data are shown as means ± SD from 3 wells, representative of 2 independent experiments. Red dashed line indicates background level of mKO signal. The numerical data underlying the graph shown in this figure are provided in S1 Data. CFU, colony-forming unit; mKO, monomeric Kusabira Orange; Mtb, *Mycobacterium tuberculosis*.
(TIF)

S2 Fig C6 does not alter phagosome maturation characteristics independent of infection. Carboxyfluorescein (A; pH readout), DQ-BSA/AF594 (B; proteolysis readout), or BAC/AF594 (C; [Cl^−^] readout) beads were added to murine BMDMs, treated with 10 μM C6 or DMSO as a carrier control, and fluorescence tracked over time with a microplate reader. Sensor beads were also added to wells containing only media, with no macrophages (“beads only, no cells”). Data are shown as means ± SD from 2–5 wells, representative of 3 independent experiments. The numerical data underlying the graphs shown in this figure are provided in S1 Data. BAC, 10,10′-bis[3-carboxylpropyl]-9,9′-biacridinium; BMDM, bone marrow–derived macrophage.
(TIF)

S3 Fig Accumulation of analogs JSF-4271 and JSF-4300 in Mtb and host macrophages. (A) Intrabacterial accumulation of the analogs JSF-4271 and JSF-4300 versus C6. Mtb were grown in 7H9 (pH 7) ± 250 mM NaCl for 6 days, before 24-h exposure to 10 μM C6, JSF-4271, or JSF-4300, and analysis of intrabacterial compound content. (B) Intracellular accumulation of the analogs JSF-4271 and JSF-4300 versus C6. J774 cells were exposed to 10 μM C6, JSF-4271, or JSF-4300 for 24 hours, before analysis of the samples for intracellular compound content. (C) Compound accumulation during Mtb infection of J774 cells. J774 cells were infected with Mtb for 5 days, before treatment with 10 μM of C6, JSF-4271, or JSF-4300 for 24 hours, sample collection and analysis for total compound accumulation (within both J774 cells and bacteria within the J774 host cells). For (A–C), data are shown as means ± SD from triplicate wells, representative of 2 independent experiments. *p*-values were determined by two-way (A) or one-way (B and C) ANOVA with Bonferroni post hoc test for all assays. ns *p* > 0.05, **p < 0.01, ***p < 0.001. The amount of accumulated compound as the number of moles was normalized by the cell number (Mtb or J774) prior to compound incubation. The numerical data underlying the graphs shown in this figure are provided in S1 Data. Mtb, *Mycobacterium tuberculosis*; ns, not significant.
(TIF)

S1 Table Primary and secondary screen assay results for validated compound hits. (XLSX)

S2 Table List of compounds that inhibited Mtb growth in J774 cells >20% at 20 μM. (XLSX)

S1 Data Numerical data underlying the presented graphs. Excel file with numerical data underlying graphed data presented in all figure files.
(XLSX)

## Abbreviations

AUC: area under the curve
BAC: 10,10′-bis[3-carboxylpropyl]-9,9′-biacridinium
BMDM: bone marrow–derived macrophage
CMC: carboxymethyl cellulose
HPLC: high-performance liquid chromatography
LC–MS: liquid chromatography–mass spectrometry
mKO: monomeric Kusabira Orange
MS/MS: tandem mass spectrometry
Mtb: *Mycobacterium tuberculosis*
PBS: phosphate buffered saline
PFA: paraformaldehyde
PK: pharmacokinetic
qRT-PCR: quantitative real-time PCR
RT: room temperature
SAR: structure–activity relationship
