# Supplementary figures and images for: Targeting Mycobacterium tuberculosis response to environmental cues for the development of effective antitubercular drugs
Source: PLoS Biol. 2021 Jul 28;19(7):e3001355. doi: 10.1371/journal.pbio.3001355 (PMC8351955; doi:10.1371/journal.pbio.3001355)

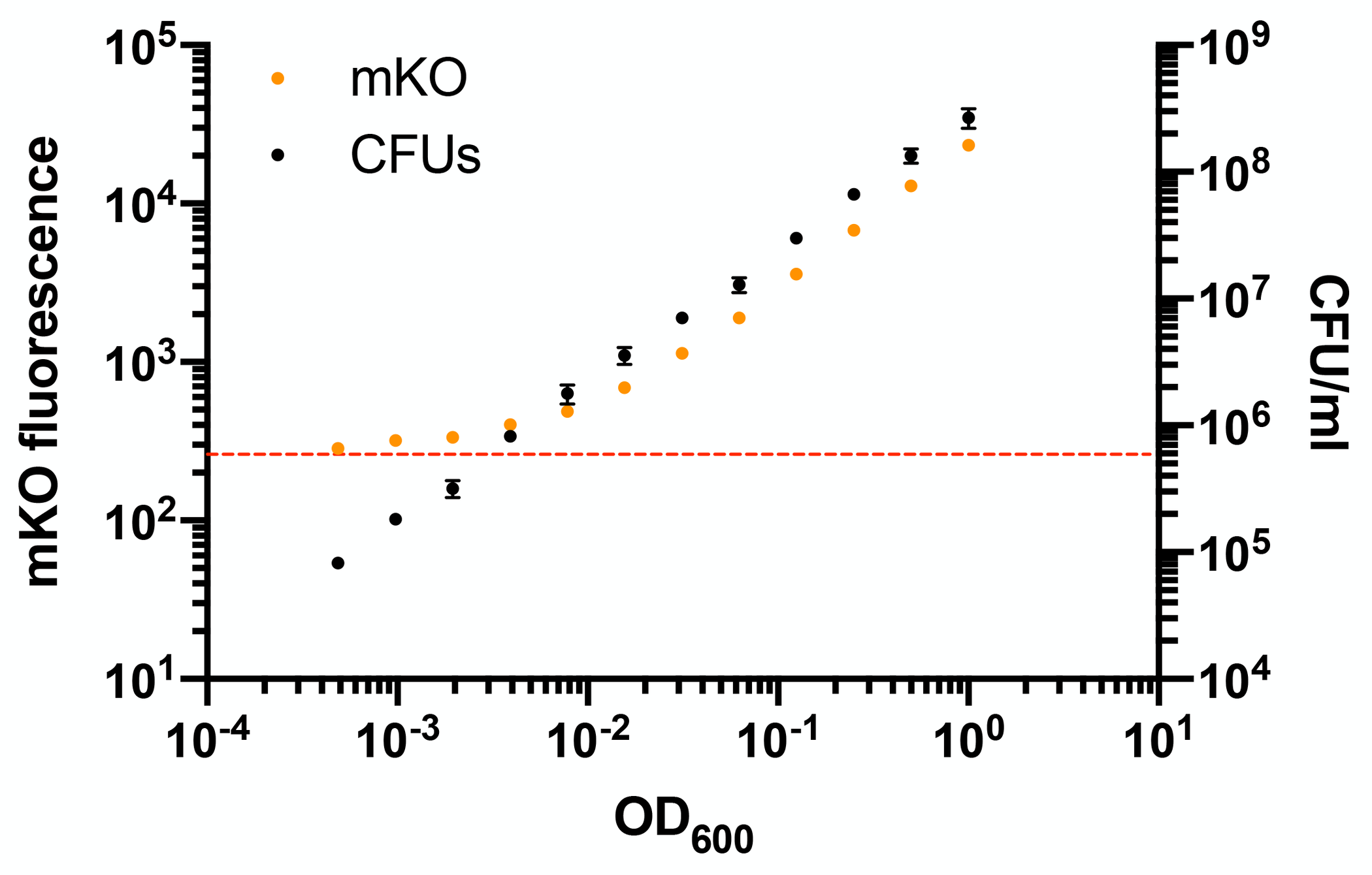

Supplement: S1 Fig — Log-phase Mtb constitutively expressing mKO was resuspended at an OD600 = 1 in 7H9 (pH 7) and diluted in 2-fold steps. mKO fluorescence was read on a plate reader (left y-axis, orange data points), before samples serial diluted and plated on 7H10 agar plates for corresponding enumeration of CFUs (right y-axis, black data points). Data are shown as means ± SD from 3 wells, representative of 2 independent experiments. Red dashed line indicates background level of mKO signal. The numerical data underlying the graph shown in this figure are provided in S1 Data. CFU, colony-forming unit; mKO, monomeric Kusabira Orange; Mtb, Mycobacterium tuberculosis. (TIF) [file pbio.3001355.s001.tif]

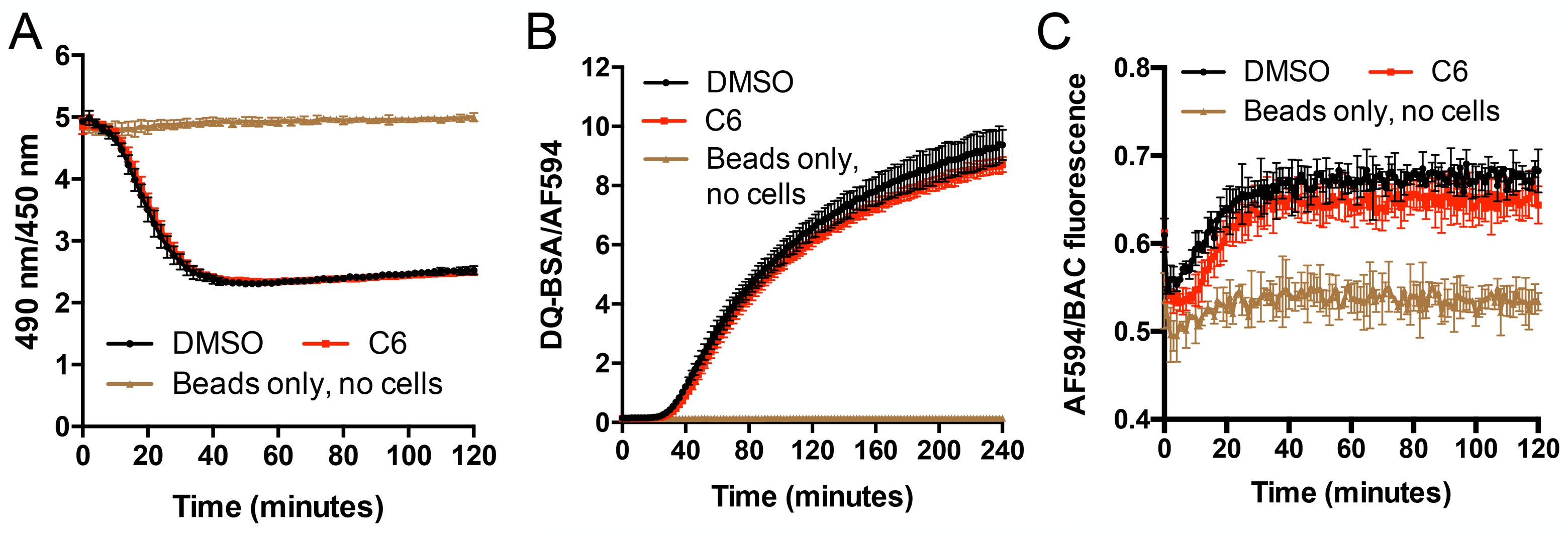

Supplement: S2 Fig — Carboxyfluorescein (A; pH readout), DQ-BSA/AF594 (B; proteolysis readout), or BAC/AF594 (C; [Cl−] readout) beads were added to murine BMDMs, treated with 10 μM C6 or DMSO as a carrier control, and fluorescence tracked over time with a microplate reader. Sensor beads were also added to wells containing only media, with no macrophages (“beads only, no cells”). Data are shown as means ± SD from 2–5 wells, representative of 3 independent experiments. The numerical data underlying the graphs shown in this figure are provided in S1 Data. BAC, 10,10′-bis[3-carboxylpropyl]-9,9′-biacridinium; BMDM, bone marrow–derived macrophage. (TIF) [file pbio.3001355.s002.tif]

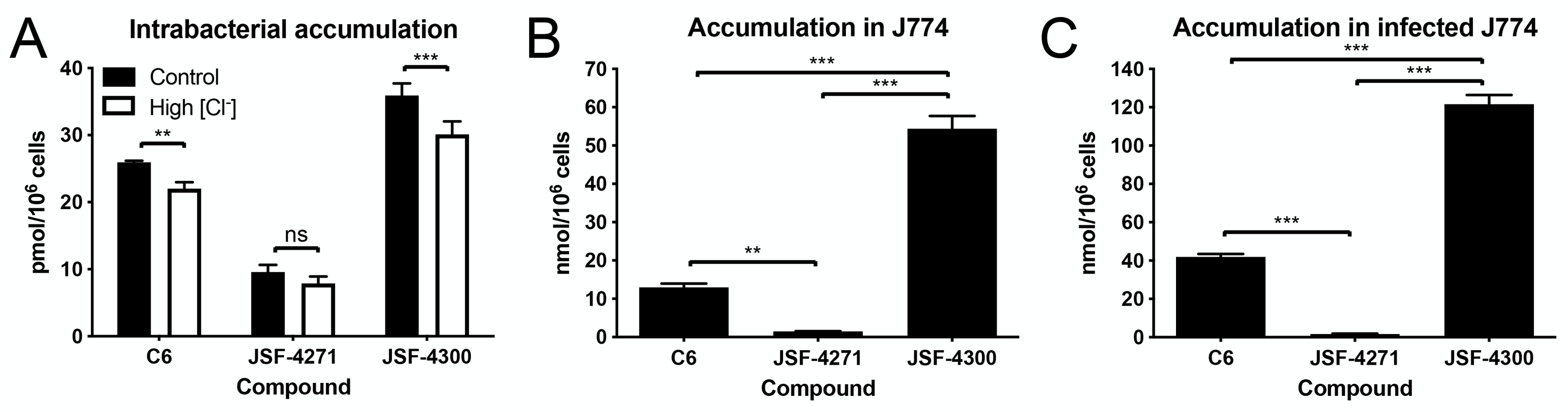

Supplement: S3 Fig — (A) Intrabacterial accumulation of the analogs JSF-4271 and JSF-4300 versus C6. Mtb were grown in 7H9 (pH 7) ± 250 mM NaCl for 6 days, before 24-h exposure to 10 μM C6, JSF-4271, or JSF-4300, and analysis of intrabacterial compound content. (B) Intracellular accumulation of the analogs JSF-4271 and JSF-4300 versus C6. J774 cells were exposed to 10 μM C6, JSF-4271, or JSF-4300 for 24 hours, before analysis of the samples for intracellular compound content. (C) Compound accumulation during Mtb infection of J774 cells. J774 cells were infected with Mtb for 5 days, before treatment with 10 μM of C6, JSF-4271, or JSF-4300 for 24 hours, sample collection and analysis for total compound accumulation (within both J774 cells and bacteria within the J774 host cells). For (A–C), data are shown as means ± SD from triplicate wells, representative of 2 independent experiments. p-values were determined by two-way (A) or one-way (B and C) ANOVA with Bonferroni post hoc test for all assays. ns p > 0.05, **p < 0.01, ***p < 0.001. The amount of accumulated compound as the number of moles was normalized by the cell number (Mtb or J774) prior to compound incubation. The numerical data underlying the graphs shown in this figure are provided in S1 Data. Mtb, Mycobacterium tuberculosis; ns, not significant. (TIF) [file pbio.3001355.s003.tif]
